# Effect of Phenolic Extract from Red Beans (*Phaseolus vulgaris* L.) on T-2 Toxin-Induced Cytotoxicity in HepG2 Cells

**DOI:** 10.3390/foods11071033

**Published:** 2022-04-02

**Authors:** Carmen Martínez-Alonso, Mercedes Taroncher, Luigi Castaldo, Luana Izzo, Yelko Rodríguez-Carrasco, Alberto Ritieni, María-José Ruiz

**Affiliations:** 1 Department of Preventive Medicine and Public Health, Food Science, Toxicology and Forensic Medicine, Faculty of Pharmacy, University of Valencia, Burjassot, 46100 Valencia, Spain; carmar36@alumni.uv.es (C.M.-A.); mercedes.taroncher@uv.es (M.T.); m.jose.ruiz@uv.es (M.-J.R.); 2 Department of Pharmacy, Faculty of Pharmacy, University of Naples “Federico II”, Via Domenico Montesano 49, 80131 Naples, Italy; luigi.castaldo2@unina.it (L.C.); luana.izzo@unina.it (L.I.); alberto.ritieni@unina.it (A.R.)

**Keywords:** beans, phenolic compounds, T-2 toxin, HepG2 cells, reactive oxygen species

## Abstract

Red beans contain human bioactive compounds such as polyphenols. Several in vitro studies have proposed the natural compounds as an innovative strategy to modify the toxic effects produced by mycotoxins. Hence, in this work, a complete investigation of the polyphenolic fraction of red beans was performed using a Q-Orbitrap high-resolution mass spectrometry analysis. Notably, epicatechin and delphinidin were the most detected polyphenols found in red bean extracts (3.297 and 3.108 mg/Kg, respectively). Moreover, the red bean extract was evaluated against the T-2 toxin (T-2) induced cytotoxicity in hepatocarcinoma cells (HepG2) by direct treatment, simultaneous treatment, and pre-treatment assays. These data showed that T-2 affected the cell viability in a dose-dependent manner, as well as observing a cytotoxic effect and a significant increase in ROS production at 30 nM. The simultaneous treatment and the pre-treatment of HepG2 cells with red bean extract was not able to modify the cytotoxic T-2 effect. However, the simultaneous treatment of T-2 at 7.5 nM with the red bean extract showed a significant decrease in ROS production, with respect to the control. These results suggest that the red bean extract could modulate oxidative stress on HepG2 cells.

## 1. Introduction

Red beans (*Phaseolus vulgaris* L.), in addition to being a great source of vegetable protein, fiber, and certain micronutrients in the human diet, contain a great variety of bioactive compounds. Bioactive compounds are simple substances that have biological activity, associated with their ability to modulate one or more metabolic processes, which results in the promotion of better health conditions. Different bioactive compounds have been studied for their positive effect on human health, such as the following: enzymes, probiotics, prebiotics, fibers, phytosterols, peptides, proteins, saponins, unsatured fatty acids, and phenolic compounds, among others [1]. Particularly, colored beans have significant amounts of phenolic compounds. Phenolic compounds are classified as flavonoids (flavones, flavonols, flavanones, isoflavones, anthocyanins, chalcones, dihydrochalcones, and catechins), phenolic acids (hydroxybenzoic, hydroxyphenyl acetic, hydroxyphenyl pentanoic, and cinnamic hydroxyl acids), tannins, stilbenes, and lignans. Phenolic acids, flavonoids, and anthocyanidins are the main phenolic compounds identified and characterized in beans [2,3]. Phenolic compounds determine the color of the seeds of these legumes; hence, in general, a higher phenolic content is observed in more pigmented beans [4]. Phenolic compounds, besides from contributing to the smell, taste, and color of food, have a long-term intake that could play a bioactive role due to their antioxidant activity, which has been related to the prevention of obesity, cardiovascular and neurodegenerative diseases, cancer, and diabetes, as well as exhibiting anti-inflammatory, antimutagenic, and antibacterial properties [5,6]. Madhujith et al. demonstrated that beans, especially those with colored skins, possess strong antioxidant activity as measured by different model systems [7]. Epidemiological studies correlated the consumption of procyanidin-rich foods with a lower incidence of inflammatory disease and diseases of multifactorial pathogenesis [8]. Similarly, the transcription and secretion of proinflammatory cytokines, including IL-1β, IL-2, IL-6, TNF-α, and interferon-γ, could be down-regulated by procyanidins, as reported in some in vitro and in vivo studies [9,10].

Despite the fact that legumes are protein rich foods, they are lacking in sulphur-containing amino acids. On the other hand, cereals contain sulphur amino acids but are limited in the essential amino acid lysine. Hence, a combination of legumes and cereals would improve the protein and nutrient density of the subsequent food products. However, the resulting high nutritional value food products could be susceptible to deterioration by fungal contamination, accompanied by the production of mycotoxins [11].

Mycotoxins are the secondary metabolites produced by filamentous fungi. The species assigned to the *Aspergillus*, *Penicillium*, *Alternaria*, *Claviceps*, and *Fusarium* genera produce a wide range of mycotoxins, which can contaminate food and feed, resulting in a significant threat to human and animal health [12]. Trichothecenes are a complex group of tetracyclic sesquiterpenoids produced by several *Fusarium* spp. Type A and B trichothecenes are the most relevant mycotoxins reported in food and feed regarding their incidence and concentration [13]. Among the trichothecenes, T-2 toxin (T-2) is the compound that shows the highest toxicity. Several studies have reported that T-2 toxin causes multiple damages to organs such as the kidney, liver, brain, gastrointestinal tract, and bone marrow [14]. Similarly, the toxic effects derived from repeated exposure to T-2 include genotoxicity, immunotoxicity, neurotoxicity, and reproductive toxicity [15,16]. Therefore, the European Food Safety Authority (EFSA) established a tolerable daily intake (TDI) for T-2 and its main metabolite HT-2 of 0.02 µg/Kg body weight (bw) to limit their exposure [13].

Based on the abovementioned information, the aims of this work are as follows: (i) to evaluate the total phenolic content (TPC), the phenolic profile, using ultra-high-performance liquid chromatography coupled with high-resolution mass spectrometry (UHPLC-Q-Orbitrap HRMS), and the antiradical activity of red bean extracts, and (ii) to assess the effect of the red bean extract on T-2 toxin-induced cytotoxicity in human hepatocarcinoma (HepG2) cells.

## 2. Materials and Methods

### 2.1. Chemicals and Reagents

Methanol (MeOH) and ethanol (EtOH) of HPLC grade were acquired from Merck (Darmstadt, Germany). Formic acid and ammonium formate were obtained from Fluka (Milan, Italy). Ethyl acetate was purchased from Merck Life Science S.L. (Madrid, Spain). Deionized water (resistivity < 18 MΩ cm) was obtained using a Milli-Q water purification system (Millipore, Bedford, MA, USA).

The chemical reagents and cell culture components used, namely Dulbecco’s Modified Eagle’s Medium (DMEM), penicillin, streptomycin, trypsin/EDTA solutions, phosphate buffered saline (PBS), Newborn Calf Serum (NBCS), methylthiazoltetrazolium salt (MTT) dye, dimethyl sulfoxide (DMSO), Sorensen’s glycine buffer, dichlorodihydrofluorescein diacetate (H_2_-DCFDA), 2,2-diphenyl-1-picrylhydrazyl (DPPH), Folin–Ciocalteu′s phenol reagent, gallic acid (C_7_H_6_O_5_), potassium chloride (KCl), sodium hydroxide (NaOH), hydrochloric acid (HCl), sodium chloride (NaCl), sodium phosphate dibasic (Na_2_HPO_4_), potassium phosphate monobasic (KH_2_PO_4_), and sodium carbonate (Na_2_CO_3_), were acquired from Sigma-Aldrich (Barcelona, Spain).

The standard of T-2 (MW: 466.52 g/mol) was purchased from Sigma-Aldrich (Barcelona, Spain). Standards of polyphenols (purity > 98%), namely protocatechuic acid, cyanidin 3,5-diglucoside, epicatechin, chlorogenic acid, cyanidin 3-galactoside, caffeic acid, catechin, *p*-cumaric acid, apigenin 7-glucoside, genistein, delphinidin, naringin, cyanidin, rosmarinic acid, myricitrin, diosmin, isoquercetin, rutin, kaempferol 3-glucoside, vitexin, ellagic acid, luteolin 7-glucoside, myricetin, diadzein, quercetin, delphinidin 3,5-diglucoside, naringenin, luteolin, kaempferol, and apigenin, were acquired from Sigma-Aldrich (Milan, Italy). Stock solutions of T-2 were prepared in MeOH at appropriate working concentrations and maintained in the dark at −20 °C.

### 2.2. Preparation of Red Bean Extract

The polyphenols were extracted from red beans according to the procedure reported in the literature with some modifications [17]. In summary, 0.5 g of ground beans was extracted with 10 mL of a solution MeOH:H_2_O. The assayed mixtures were 20:80, 30:70, 50:50, 70:30, and 80:20 (***v*/*v***). Hydrochloric acid 2N was added to the sample until a pH adjustment to 2, to avoid the protonated forms of carboxylic groups present in polyphenols. Then, the mixture was subjected to a horizontal shaker (250 rpm) at room temperature for 3 h and centrifuged for 3 min at 3500 rpm. Finally, MeOH was evaporated from the acidified sample extract under reduced pressure (250 mbar) at 50 °C for 15 min by Buchi Rotavapor R-200 (Buchi, Postfach, Switzerland) and the aqueous extract containing polyphenols was filtered with a 0.2 μm polytetrafluoroethylene (PTFE) filter and stored in amber glass flask at 4 °C.

### 2.3. Determination of Total Phenolic Content (TPC)

The Folin–Ciocalteu assay was used to determine the total phenolic content in accordance with the procedure reported by Izzo et al. [18]. Briefly, 0.5 mL of red bean extract or blank (deionized water) was diluted with deionized water (4.5 mL) and 0.25 mL of Folin–Ciocalteu reagent 1 N was added. Then, 1 mL of 2% sodium carbonate solution was added and the mixture was allowed to stand at room temperature for 1 h in dark conditions. Finally, the absorbance was measured with a spectrophotometer at 765 nm against a reagent blank. The analysis was carried out in triplicate and the results were expressed as mg of gallic acid equivalents (GAE) per Kg of sample.

### 2.4. Determination of Polyphenolic Profile

The polyphenolic profile of the red bean extracts was carried out on a UHPLC-Q Exactive Orbitrap-HRMS system (Thermo Fisher Scientific, Waltham, MA, USA), composed of a Dionex Ultimate 3000 liquid chromatograph equipped with a solvent rack compartment (SRD-3x00), a quaternary rapid separation pump (LPG-3400RS), a rapid separation autosampler (WPS-3000RS), and a temperature-controlled column compartment (TCC-3000SD). The chromatographic separation was performed on a Kinetex F5 (50 × 2.1 mm; 1.7 µm) reverse-phase column (Phenomenex, Milan, Italy) at 25 °C. The mobile phase consisted of water containing 0.1% formic acid (A) and MeOH containing 0.1% formic acid (B). The separation gradient consisted of an initial 0% of phase B, increasing to 40% B in 1 min, 80% B in a further 1 min, and 100% B in 3 min. Then, the gradient was held at 100% B for 4 min and reduced to 0% B in 2 min, followed by 2 min of column re-equilibration at 0% B. The total run time was 13 min. The flow rate was 0.5 mL/min and the injection volume was 1 µL.

The mass spectrometer was equipped with an electrospray (ESI) source that simultaneously operates in positive and negative ion switching mode. Full ion MS and all ion fragmentation (AIF) were set as scan events. The following settings were used in full MS mode: resolution power of 70,000 Full Width at Half Maximum (FWHM) (defined for *m*/*z* 200); automatic gain control (AGC) target: 1 × 10^6^; scan range: 80–1200 *m*/*z*; injection time set to 200 ms; scan rate set at 2 scan/s. The ion source parameters were as follows: sheath gas pressure: 18; auxiliary gas: 3; spray voltage: 3.5 kV; capillary temperature: 320 °C; S-lens RF level: 60; auxiliary gas heater temperature: 350 °C. For the scan event of AIF, the parameters in the negative and positive mode were set as follows: mass resolving power = 17,500 FWHM; ACG target = 1 × 10^5^; maximum injection time = 200 ms; scan time = 0.10 s; scan range = 80–1200 *m*/*z*; isolation window to 5.0 *m*/*z*; retention time to 30 s. The collision energy was varied between 10 and 60 eV to acquire representative product ion spectra.

For the identification and confirmation of the molecular ion and fragments, a mass tolerance below 5 ppm was set. Data analysis and processing were performed using Xcalibur software, v. 3.1.66.10 (Xcalibur, Thermo Fisher Scientific, Waltham, MA, USA).

### 2.5. Determination of Antiradical Activity (DPPH)

The total free radical scavenging activity of the red bean extracts was determined using the method reported in the literature with modifications [19]. Briefly, DPPH (4 mg) was solubilized in 10 mL of MeOH and then diluted to reach an absorbance value of 0.90 (±0.05) at 517 nm. This solution was used to perform the assay and 200 µL of red bean extract was added to 1 mL of working solution. The mixture was vortexed, kept for 90 min in the dark, and centrifuged for 5 min at 11,000 rpm. Finally, the decreased absorbance was measured at 517 nm. The analysis was carried out in triplicate and the results were expressed as mmol Trolox Equivalents (TE) per Kg of sample.

### 2.6. Cell Culture

Human hepatocarcinoma (HepG2) cells (ATCC: HB-8065) were cultured in DMEM medium supplemented with 10% NBCS, 100 U/mL penicillin, and 100 mg/mL streptomycin. The cells were maintained at pH 7.4, 5% CO_2_ at 37 °C, and 95% air atmosphere at constant humidity. The cells were subcultured routinely twice a week with only a small number of sub-passages (<20 passages) in order to maintain genetic homogeneity. HepG2 cells were subcultured after trypsinization in a 1:3 split ratio. The medium was changed every 5 days. The final mycotoxin concentrations tested were achieved by adding T-2 mycotoxin to the culture medium, with a final MeOH concentration ≤ 1% (*v*/*v*).

### 2.7. HepG2 Cells Treatment

The HepG2 cells were cultured in 96-well tissue culture plates by adding 200 μL/well of density at 2 × 10^4^ cells/well. After the cells reached 80% confluence, the culture medium was replaced with a fresh medium containing different concentrations of T-2 (7.5, 15, and 30 nM) and serial dilutions of red bean extracts (from 1:32 to 1) buffered to pH 7.4. Then, the plates were incubated in the dark at 37 °C and 5% CO_2_ for 24 h. The assayed T-2 concentrations correspond to sublethal T-2 concentrations for HepG2 cells (<IC_50_), based on previous studies carried out in our laboratory [20], and they were IC_50_/2, IC_50_/4, and IC_50_/8, respectively.

The following two more assays were performed: simultaneous treatment and pre-treatment. On one hand, for pre-treatment studies, HepG2 cells were exposed to one red bean extract dilution according to previous cell proliferation assays (red bean extract 1:8 dilution) for 1 and 24 h. Then, the medium containing the red bean extract was removed and cells were exposed at the T-2 concentrations described above for 24 h. On the other hand, to conduct studies of simultaneous treatment, HepG2 cells were exposed to the assayed T-2 concentrations and the 1:8 diluted red bean extract for 24 h.

Appropriate controls containing the same amount of solvent were included in each experiment.

### 2.8. Determination of Cell Viability

Cell viability was determined in HepG2 cells by the MTT assay. The MTT assay is based on the capacity of viable cells to metabolize, via a mitochondrial-dependent reaction, specifically, the reduction of yellow tetrazolium salt to an insoluble purple formazan crystal. The MTT assay was carried out according to the procedure reported by Ruiz et al. [21]. In summary, after treatment studies, the medium containing the compounds was removed and each well received 200 μL of fresh medium containing 50 μL of MTT. The plates were returned to the incubator in the dark at 37 °C and 5% CO_2_ for 3 h. Then, the MTT solution was removed and 200 μL of DMSO was added, followed by 25 μL of Sorensen’s glycine buffer. The absorbance was measured at 620 nm on a Wallace Victor^2^, model 1420 multilabel counter (PerkinElmer, Turku, Finland). The blank absorbance value (from wells without cells but treated with MTT) was subtracted from all absorbance values.

Cell viability was expressed as a percentage relative to control cells (≤1% MeOH). Three independent experiments were conducted with eight replicates each, and the results were expressed as the mean ± standard error of the mean (SEM) of different independent experiments.

### 2.9. Determination of Reactive Oxygen Species (ROS)

Intracellular ROS production was monitored in HepG2 cells by adding H_2_-DCFDA [22]. H_2_-DCFDA is taken up by cells and then deacetylated by intracellular esterases; the resulting non-fluorescent 2′,7′-dichlorodihydrofluorescein (H_2_-DCF) is converted to greatly fluorescent dichlorofluorescein (DCF) when oxidized by ROS. Briefly, 2 × 10^4^ cells/well were seeded in a 96-well black polystyrene culture microplate. Once cells exhibited 80% confluence, the culture medium was replaced and cells were loaded with 20 µM H_2_-DCFDA in a fresh medium for 20 min in darkness. Then, H_2_-DCFDA was removed and replaced by a fresh medium containing 1:8 diluted red bean extract, T-2 (30, 15, and 7.5 nM), and the combination of diluted red bean extract with T-2 at the different concentrations assayed. Finally, the fluorescence emitted by the DCF was monitored at different times (0, 5, 15, 30, 45, 60, 90, and 120 min) on a Wallace Victor^2^, model 1420 multilabel counter (PerkinElmer, Turku, Finland), at excitation/emission wavelengths of 485/535 nm, respectively. The blank absorbance value (from wells without cells but treated with H_2_-DCFDA) was subtracted from all absorbance values.

The determinations were performed in three independent experiments with 24 replicates each and the results are expressed as an increase (%) in fluorescence in respect to control cells.

### 2.10. Statistical Analysis

Statistical analysis of data was carried out using the Statgraphics version 16.01.03 statistical package (IBM Corp., Armonk, NY, USA). Data are expressed as mean ± SEM of different independent experiments. The statistical analysis of the results was performed by Student’s *t*-test for paired samples. The differences between groups were analyzed by employing one-way analysis of variance (ANOVA) continued by the Tukey HDS *post-hoc* test for multiple comparisons. Statistical significance was considered for *p* ≤ 0.05.

## 3. Results and Discussion

### 3.1. Total Phenolic Content and Antiradical Activity of Red Bean Extract

The extraction of phenolic compounds from red beans was optimized by testing different mixtures of MeOH:H_2_O and further determined through the Folin–Ciocalteu assay. The results are shown in Table 1. Based on the results obtained, the mixture 70:30 was optimal to extract phenolic compounds from red beans. The obtained TPC content (2325 ± 98 mg GAE/Kg) was in the same range to that of other studies performed on similar food matrices with values of 1230 mg/Kg [23], 1205 mg/Kg [24], 1690–4850 mg/Kg [25], and 3450 mg/Kg [26]. The TPC and DPPH data of undiluted red bean extracts are shown in Table S1.

The total free radical scavenging activity of the red bean extract was evaluated through the DPPH assay. The results are in agreement with the previous assay being the highest antioxidant activity obtained with the mixture MeOH:H_2_O (70:30, *v*/*v*) (Table 1). In addition, it was observed that the larger the phenolic content, the better the free radical scavenging activity. Significant differences in the phenolic content and the antioxidant activity amongst legume extracts were also reported by Zhao et al. [27]. Similarly, the values of antiradical activity obtained from red bean extract (49.2 ± 4.6 mmol TE/Kg) were comparable to those reported in other studies [28,29].

Besides the antioxidant properties shown by the phenolic compounds, the anti-inflammatory activity by different mechanisms, including modulation of the inflammatory cascade, has also been reported in the literature [30]. García-Lafunete et al. reported that the phenolic rich extracts from beans inhibited the expression of IL-1β, IL-6, and TNF-α genes of stimulated macrophages RAW 246.7, with colored beans showing more activity than white beans [31].

### 3.2. Identification and Quantification of Active Compounds in Red Bean Extract

A total of 30 polyphenols were investigated in the red beans by using UHPLC-Q-Orbitrap HRMS analysis. The chromatographic and spectrometric parameters are shown in Table 2. The results showed a good chromatographic shape and separation of all studied compounds through the UHPLC gradient system employed within a 13 min run. Four different structural isomers, namely genistein and apigenin (*m*/*z* 269.0455), and vitexin and apigenin 7-glucoside (*m*/*z* 431.0983), were found. Compound identification was conducted by comparing the retention times of the standards with the peaks observed in sample extracts. For quantification purposes, calibration curves at eight concentration levels were built in triplicate. All regression coefficients were greater than 0.990.

The quantification of the main phenolic acids and flavonoids in red beans was performed by using a UHPLC-Q-Orbitrap HRMS method. The results are shown in Table 3. Up to 22 polyphenols were detected. Among the quantified polyphenols, epicatechin was the major flavanol (3.297 ± 0.119 mg/Kg). The delphinidin, an important anthocyanidin of pigmented beans, was quantified at 3.108 ± 0.023 mg/Kg. Flavanols and anthocyanidins represented 36% and 24.5% of total polyphenolic compounds in red bean samples, respectively. The other important polyphenols quantified in samples were *p*-coumaric acid, isoquercetin, and kaempferol 3-O-glucoside. The polyphenols content of undiluted red bean extracts are shown in Table S2.

### 3.3. Effects of Red Bean Extract on the Cell Viability by Individual Exposure

**Figure 1:**
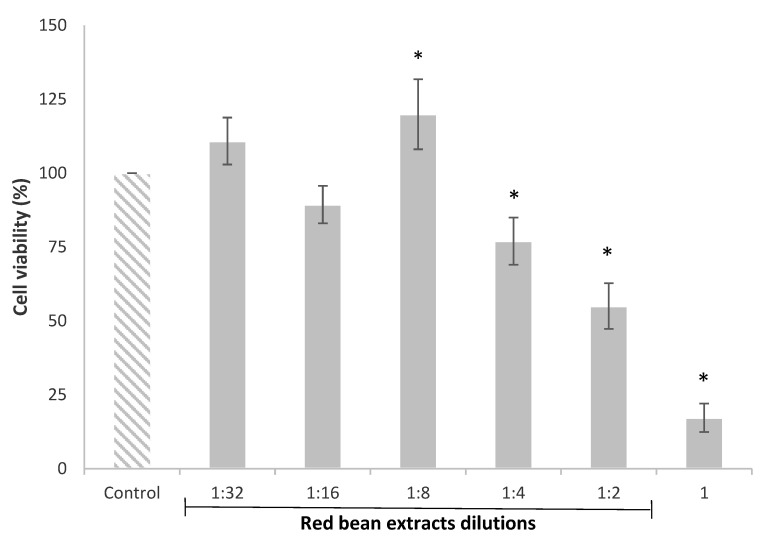
HepG2 cell viability after 24 h of treatment with red bean extracts (undiluted extract (1) and dilutions from 1:2 to 1:32). All values are expressed as mean ± SEM of 3 replicates. (*) *p* ≤ 0.05 indicates significant differences compared to control.

The HepG2 cell viability evaluated by the MTT assay after 24 h of exposure with red bean extract dilutions, from 1 to 1:32, is shown in Figure 1. The results clearly indicated that the viability of HepG2 cells was affected by the more concentrated red bean extracts. In particular, the undiluted (1) and diluted (1:2 and 1:4) red bean extracts significantly decreased HepG2 cell viability from 83% to 23%. Nonetheless, the red bean extract diluted 1:8 significantly increased the cell viability (20%) compared to the control.

The reduction in cell viability caused by the more concentrated or undiluted extracts has also been observed in other studies. Recently, Ziemlewska et al. [32] indicated that high concentrations of the bioactive compounds contained in red fruits inhibited the cell cycle in the G2/M phase and caused cell death, exerting a negative impact on cell viability. These authors demonstrated that high concentrations of phenolic extracts from berries induced apoptosis due to the activation of the caspases.

### 3.4. Effects of Simultaneous Treatment in HepG2 Cell Viability Exposed to T-2 and Red Bean Extract

**Figure 2:**
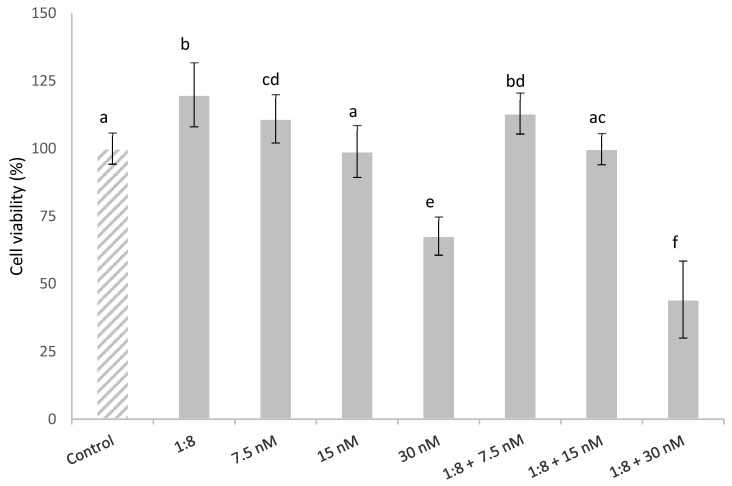
HepG2 cell viability (%) during 24 h of exposure to T-2 (7.5, 15 and 30 nM) and 1:8 diluted red bean extract alone and in combination. All values are expressed as mean ± SEM of 3 replicates. Values in the same figure with different superscript letters are significantly different (*p* ≤ 0.05). 1:8 = 1:8 diluted red bean extract.

The effect in HepG2 cells simultaneously exposed to T-2 (7.5, 15 and 30 nM) and 1:8 diluted red bean extract is described in Figure 2. The HepG2 cell viability was affected in a concentration-dependent manner. Cytotoxic effects were observed in the cells exposed to 30 nM T-2 alone and in combination with the extract, with a significant reduction in cell viability compared to the control cells by 32% and 56%, respectively. This could be due to the fact that the diluted red bean extract did not completely prevent T-2 cytotoxicity. The diluted red bean extract slightly lowered HepG2 cell viability, which was a statistically significant decrease only in cells exposed to the highest T-2 concentration; whereas it did not completely prevent T-2 cytotoxicity.

On the other hand, cells exposed to the lowest T-2 concentration tested (7.5 nM) showed a significant increase in cell viability (11%) compared to the control; cell viability was slightly higher (13%) in cells simultaneously treated with T-2 and the diluted red bean extract.

### 3.5. Effects of Pre-Treatment in HepG2 Cell Viability Exposed to T-2 and Red Bean Extract

**Figure 3:**
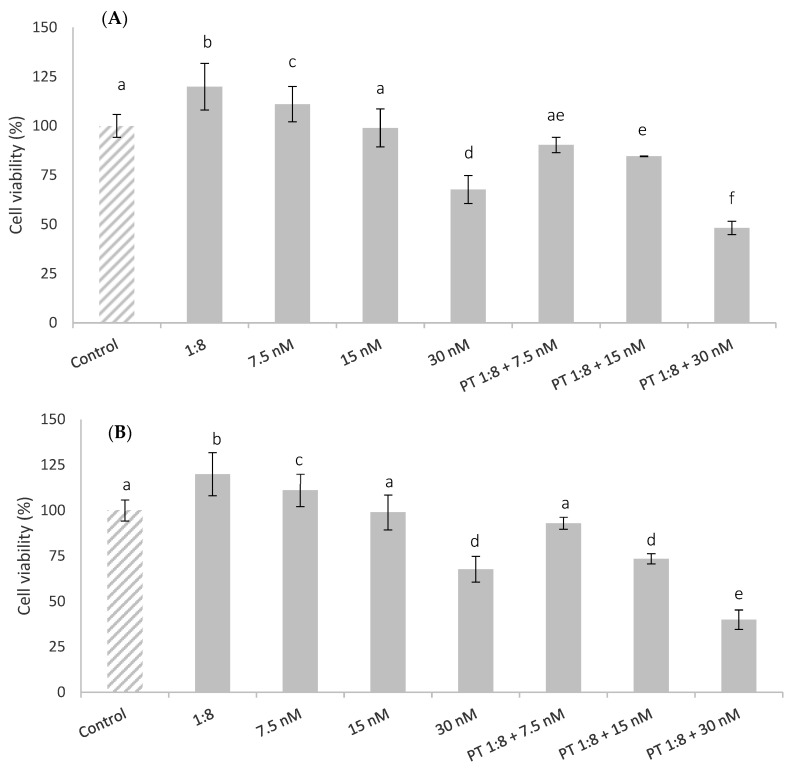
HepG2 cell viability (%) of pre-treated cells (PT) with 1:8 red bean extract for 1 h (**A**) and 24 h (**B**) and afterward exposed to T-2 (7.5, 15, and 30 nM) during 24 h. All values are expressed as mean ± SEM of 3 replicates. Values in the same figure with different superscript letters are significantly different (*p* ≤ 0.05).

The effects of cell pre-treatment with red bean extract for 1 h and 24 h before the T-2 addition are shown in Figure 3. The results indicated that the pre-treatment with red bean extract was not able to protect or ameliorate the cytotoxic T-2 effect in HepG2 cells in all T-2 concentrations tested. This effect was increased at 24 h of exposure. Similar results were reported by Kössler et al. [33], who showed that curcumin (phenolic compound) reduced cell viability and induced apoptosis in human embryonic kidney cells (HEK293) in a dose-dependent manner at 22 h of exposure. There are no other data from in vitro studies by other authors with bean extracts. However, Vila-Donat et al. [34] reported synergistic cytoprotective effects against cytotoxicity induced by alternariol on Caco-2 cells exposed to an extract obtained from other types of legumes, such as lentils.

### 3.6. Reactive Oxygen Species

**Figure 4:**
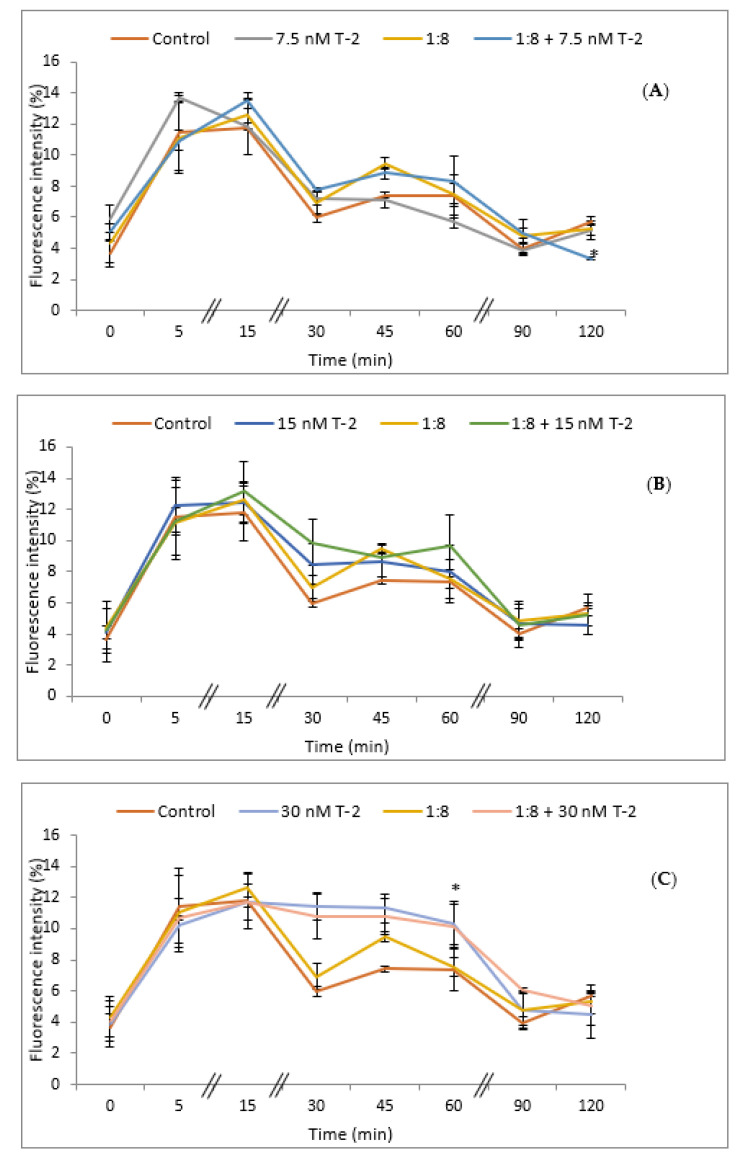
Intracellular ROS production in HepG2 cells exposed to: (**A**) 7.5 nM T-2; (**B**) 15 nM T-2; (**C**) 30 nM T-2; and 1:8 diluted red bean extract, alone or in combination, after 0, 5, 15, 30, 45, 60, 90, and 120 min. All values are expressed as mean ± SEM of 4 replicates. (*) *p* ≤ 0.05 represents significant difference as compared to control values.

The intracellular accumulation of ROS in HepG2 cells exposed to T-2 toxin was analyzed using the dichlorofluorescein assay (DCFH-DA). The cells were exposed to T-2 (7.5, 15, and 30 nM) and 1:8 diluted red bean extract for different exposure times (0, 5, 15, 30, 45, 60, 90, and 120 min), and the results are expressed as an increase in fluorescence (%) with respect to the control (Figure 4). The simultaneous exposure to T-2 at the lowest concentration tested (7.5 nM) and the red bean extract showed a significant reduction (*p* ≤ 0.05) in ROS production after 120 min of exposure with respect to control cells (Figure 4A); whereas there were no significant differences in ROS production at 15 nM with respect to control cells (Figure 4B). On the contrary, a significant increase (*p* ≤ 0.05) in ROS production with respect to controls was observed in the case of the simultaneous exposure to T-2 at 30 nM alone and in combination with the red bean extract at 60 min of exposure (Figure 4C).

Data reported by Lee et al. [34] demonstrated that the treatment of HepG2 cells with extracts obtained from different types of beans (black, red, and green) against the Tert Butyl Hydroperoxide (TBHP) agent significantly decreased the production of ROS. On the other hand, Yang et al. [3] showed that the level of intracellular ROS production in HCT116 cells decreased significantly in cells treated with phenolic extracts of beans compared to untreated cells.

The abovementioned results can demonstrate the ability of phenolic extracts to modulate oxidative stress. However, further studies with regard to biological activity, including studies into the mechanisms of action and structure-activity relationships, are necessary to fully understand the modes of action of these bioactive compounds and to fully exploit their cytoprotective potential effect, as highlighted in the literature [35].

## 4. Conclusions

The chemical profile obtained from this study evidenced that red beans are a rich source of bioactive compounds such as flavanols and anthocyanidins, which confers antiradical activity and human health benefits. On the other hand, red bean extract (diluted 1:8) showed a significant increase in HepG2 cell proliferation after 24 h of exposure. Similar results were observed after HepG2 cell exposure to T-2 toxin at the lowest concentration assayed (7.5 nM) corresponding to its IC_50_/8, which could indicate a probable adaptative response of HepG2 cells. However, higher T-2 concentrations showed cytotoxic effects and ROS production on HepG2 cells. The results from the simultaneous and pre-treatment assays indicated that the 1:8 diluted red bean extract did not prevent T-2 cytotoxicity neither in simultaneous exposures nor with the pre-treatment. Finally, the combination of T-2 at 7.5 nM with the diluted red bean extract showed a decrease in ROS production compared to the control at the longest exposure time tested (120 min). The antioxidant activity or the possible T-2 hormetic effect observed in simultaneous treatment could be responsible for the latter result, suggesting that the red bean extract could modulate oxidative stress on HepG2 cells.

## Figures and Tables

**Table 1:** Total phenolic content (TPC) and antiradical activity (DPPH) of dry red beans using different mixtures of MeOH:H_2_O. Values are reported as mean ± SD of independent experiments performed in triplicate.

MeOH:H_2_O (*v*/*v*)	TPC (mg GAE/Kg ± SD)	DPPH (mmol TE/Kg ± SD)
80:20	1892 ± 237	42.3 ± 5.8
70:30	2325 ± 98	49.2 ± 4.6
50:50	1348 ± 24	30.4 ± 6.1
30:70	1272 ± 8	29.2 ± 8.7
20:80	1287 ± 28	27.5 ± 7.3

TPC: total phenolic content; GAE: gallic acid equivalents; DPPH: antiradical activity; TE: Trolox equivalents.

**Table 2:** Chromatographic and spectrometric parameters, including retention time, adduct ion, theoretical and measured mass (*m*/*z*), accuracy, and sensibility, for the investigated bioactive compounds (*n* = 30).

Compound	RT (min)	Chemical Formula	Adduct Ion	Theoretical Mass (*m*/*z*)	Measured Mass (*m*/*z*)	Product Ion	Mass Accuracy (Δ ppm)	LOD (mg/Kg)	LOQ (mg/Kg)
Protocatechuic acid	1.60	C_7_H_6_O_4_	[M − H]^−^	153.01930	153.01857	109.02840	−4.771	0.026	0.078
Cyanidin 3,5-diglucoside	3.03	C_27_H_31_O_16_	[M + H]^+^	611.16066	611.16022	449.19708–287.06469	−0.719	0.026	0.078
Epicatechin	3.08	C_15_H_14_O_7_	[M − H]^−^	289.07176	289.07202	221.94647–203.09201–161.04478	0.890	0.013	0.039
Chlorogenic acid	3.20	C_16_H_18_O_9_	[M − H]^−^	353.08780	353.08798	191.05594–84.98998	0.509	0.013	0.039
Cyanidin 3-galactoside	3.23	C_21_H_21_O_11_	[M + H]^+^	449.10784	449.10654	287.05576	−2.894	0.026	0.078
Caffeic acid	3.25	C_9_H_8_O_4_	[M − H]^−^	179.03498	179.03455	134.99960	−2.401	0.013	0.039
Catechin	3.27	C_15_H_14_O_6_	[M − H]^−^	289.07175	289.07205	247.02241–205.10712–151.03923–125.02335	1.037	0.026	0.078
*p*-cumaric acid	3.39	C_9_H_8_O_3_	[M − H]^−^	163.04001	163.03937	119.04917	−3.925	0.026	0.078
Apigenin 7-glucoside	3.45	C_21_H_20_O_10_	[M − H]^−^	431.09837	431.09875	341.10919–283.26419	0.881	0.013	0.039
Genistein	3.47	C_15_H_10_O_5_	[M − H]^−^	269.04554	269.04562	241.14435–213.14908–151.03935	0.297	0.013	0.039
Delphinidin	3.48	C_15_H_11_O_7_	[M − H]^+^	303.04992	303.04993	257.12119–137.05981	0.033	0.026	0.078
Naringin	3.54	C_27_H_32_O_14_	[M − H]^−^	579.17193	579.17185	271.15524–227.12846–161.04475	−0.138	0.013	0.039
Cyanidin	3.57	C_15_H_11_O_6_	[M + H]^+^	287.05501	287.05472	207.05879–147.07649	−0.611	0.013	0.039
Rosmarinic acid	3.58	C_18_H_16_O_8_	[M − H]^−^	359.07724	359.07742	179.05537	0.501	0.013	0.039
Myricitrin	3.59	C_21_H_20_O_12_	[M − H]^−^	463.08820	463.08701	316.02126–178.97646	−2.57	0.013	0.039
Diosmin	3.64	C_28_H_32_O_15_	[M − H]^−^	607.16684	607.16534	300.99796–284.03838	−2.471	0.013	0.039
Isoquercetin	3.65	C_21_H_20_O_12_	[M − H]^−^	463.08820	463.08853	431.09848–187.09698–174.95542	0.712	0.013	0.039
Rutin	3.65	C_27_H_30_O_16_	[M − H]^−^	609.14611	609.14673	300.99911–271.05026–255.12390	1.017	0.013	0.039
Kaempferol 3-glucoside	3.66	C_21_H_20_O_11_	[M − H]^−^	447.09195	447.09329	284.03079–255.02881–227.07033	3.000	0.013	0.039
Vitexin	3.67	C_21_H_20_O_10_	[M − H]^−^	431.09837	431.09711	341.10803–311.05457–269.13815	−2.921	0.013	0.039
Ellagic acid	3.67	C_14_H_6_O_8_	[M − H]^−^	300.99899	300.99911	245.91669–229.93712–185.01208–117.00336	0.398	0.013	0.039
Luteolin 7-glucoside	3.68	C_21_H_20_O_11_	[M − H]^−^	447.09328	447.09381	285.04028	1.185	0.013	0.039
Myricetin	3.73	C_15_H_10_O_8_	[M − H]^−^	317.03029	317.02924	178.87917–151.00217–137.02290	−3.310	0.013	0.039
Daidzein	3.75	C_15_H_9_O_4_	[M − H]^−^	253.05063	253.04977	209.96429–225.00984	−3.398	0.013	0.039
Quercetin	3.86	C_15_H_10_O_7_	[M − H]^−^	301.03538	301.03508	174.95551	−0.996	0.013	0.039
Delphinidin 3,5-diglucoside	3.87	C_27_H_31_O_17_	[M + H]^+^	628.16340	628.16385	465.10339–303.04987	0.716	0.026	0.078
Naringenin	3.91	C_15_H_12_O_5_	[M − H]^−^	271.06120	271.06110	235.92595–151.03917	−0.368	0.013	0.039
Luteolin	3.94	C_15_H_10_O_6_	[M − H]^−^	285.04046	285.04086	174.95486–89.02095	1.401	0.013	0.039
Kaempferol	4.00	C_15_H_10_O_6_	[M − H]^−^	285.04046	285.04086	93.00679	1.403	0.013	0.039
Apigenin	4.08	C_15_H_10_O_5_	[M − H]^−^	269.04554	269.04541	225.06136–117.01828	−0.483	0.013	0.039

RT: retention time; LOD: limit of detection; LOQ: limit of quantification.

**Table 3:** Polyphenols content in dry red beans. Results are expressed as mean ± SD from three independent determinations.

Compound	Content (mg/Kg) ± SD
Apigenin 7-O-glucoside	<LOQ
Catechin	<LOQ
Chlorogenic acid	0.045 ± 0.002
Cyanidin	0.677 ± 0.046
Cyanidin 3-glucoside	<LOQ
Cyanidin 3,5-diglucoside	0.171 ± 0.015
Daidzein	<LOQ
Delphinidin	3.108 ± 0.023
Delphinidin 3,5-diglucoside	0.210 ± 0.020
Ellagic acid	0.045 ± 0.001
Epicatechin	3.297 ± 0.119
Genistin	<LOQ
Isoquercetin	1.039 ± 0.119
kaempferol 3-O-glucoside	0.803 ± 0.026
Luteolin	0.021 ± 0.001
Naringenin	<LOQ
Naringin	<LOQ
*p*-coumaric acid	1.929 ± 0.106
Protocatechiuc acid	0.532 ± 0.016
Quercetin	0.292 ± 0.026
Rosmarinic acid	<LOQ
Rutin	0.479 ± 0.037

## Data Availability

The data presented in this study are available on request from the corresponding author. The data are not publicly available to preserve the privacy of the volunteers that participated in the present study.

## Supplementary Materials

The following supporting information can be downloaded at: https://www.mdpi.com/article/10.3390/foods11071033/s1, Table S1: Total phenolic content (TPC) and antiradical activity (DPPH) of undiluted red bean extracts using different mixtures of MeOH:H_2_O. Values are reported as mean ± SD of independent experiments performed in triplicate; Table S2. Polyphenols content in undiluted red bean extracts. Results are expressed as mean ± SD from three independent determinations.
