# Supplementary material for: Effect of Phenolic Extract from Red Beans (Phaseolus vulgaris L.) on T-2 Toxin-Induced Cytotoxicity in HepG2 Cells
Source: Foods. 2022 Apr 2;11(7):1033. doi: 10.3390/foods11071033 (PMC8997370; doi:10.3390/foods11071033)
Supplement: Supplementary file 1 [file foods-11-01033-s001.zip › foods-1668545-supplementary/Supplementary material.pdf]

Table S1. Total phenolic content (TPC) and antiradical activity (DPPH) of undiluted red bean extracts using different mixtures of MeOH:H_2O. Values are reported as mean ± SD of independent experiments performed in triplicate.

Mixture (v/v)	TPC (mg/mL undiluted extract)	DPPH (mmol TE/mL undiluted extract)
20:80	0.0171	7.6×10^{-4}
70:30	0.0169	8.1×10^{-4}
50:50	0.0179	8.4×10^{-4}
30:70	0.0310	1.37×10^{-3}
80:20	0.0252	1.17×10^{-3}

TPC: total phenolic content; GAE: gallic acid equivalents; DPPH: antiradical activity; TE: Trolox equivalents

Table S2. Polyphenols content in undiluted red bean extracts. Results are expressed as mean ± SD from three inde-pendent determinations.

**Compound**	**Content (mg/mL undiluted extract)**
Apigenin 7-O-glucoside	<LOQ
Catechin	<LOQ
Chlorogenic acid	7.5×10^{-6}
Cyanidin	1.1×10^{-4}
Cyanidin 3-glucoside	<LOQ
Cyanidin 3,5-diglucoside	2.8×10^{-5}
Daidzein	<LOQ
Delphinidin	5.1×10^{-4}
Delphinidin 3,5-diglucoside	3.5×10^{-5}
Ellagic acid	7.5×10^{-6}
Epicatechin	5.4×10^{-4}
Genistin	<LOQ
Isoquercetrin	1.7×10^{-4}
kaempferol 3-O-glucoside	1.3×10^{-4}
Luteolin	3.5×10^{-6}
Naringenin	<LOQ
Naringin	<LOQ
p-coumaric acid	3.2×10^{-4}
Protocatechiuc acid	8.8×10^{-5}
Quercetin	4.9×10^{-5}
Rosmarinic acid	<LOQ
Rutin	7.9×10^{-5}
